# Body size distributions of the pale grass blue butterfly in Japan: Size rules and the status of the Fukushima population

**DOI:** 10.1038/srep12351

**Published:** 2015-07-22

**Authors:** Wataru Taira, Mayo Iwasaki, Joji M. Otaki

**Affiliations:** 1 The BCPH Unit of Molecular Physiology, Department of Chemistry, Biology and Marine Science, Faculty of Science, University of the Ryukyus, Nishihara, Okinawa 903-0213, Japan

## Abstract

The body size of the pale grass blue butterfly, *Zizeeria maha*, has been used as an environmental indicator of radioactive pollution caused by the Fukushima nuclear accident. However, geographical and temporal size distributions in Japan and temperature effects on size have not been established in this species. Here, we examined the geographical, temporal, and temperature-dependent changes of the forewing size of *Z. maha argia* in Japan. Butterflies collected in 2012 and 2013 from multiple prefectures throughout Japan demonstrated an inverse relationship of latitude and forewing size, which is the reverse of Bergmann’s cline. The Fukushima population was significantly larger than the Aomori and Miyagi populations and exhibited no difference from most of the other prefectural populations. When monitored at a single geographic locality every other month, forewing sizes were the largest in April and the smallest in August. Rearing larvae at a constant temperature demonstrated that forewing size followed the temperature-size rule. Therefore, the converse Bergmann’s rule and the temperature-size rule coexist in this multivoltine species. Our study establishes this species as a useful environmental indicator and supports the idea that the size reduction observed only in Fukushima Prefecture in 2011 was caused by the environmental stress of radioactive pollution.

Butterfly biology has been a topic of interest within many disciplines, including developmental biology, evolutionary biology, ecology, behavioral biology, and environmental sciences^1^,^2^. Within these fields, nymphalid butterflies have been utilized almost exclusively, despite the differences among these disciplines. For example, the African satyrine butterfly *Bicyclus anynana* has been used extensively in molecular biological analyses^3^. The South American *Heliconius* butterflies that form mimicry circles have been the focus of genomic studies^4^. Additionally, the draft genome sequence of the North American monarch butterfly *Danaus plexippus*, famous for its migration, has already been published^5^. One reason for the intensive use of nymphalid butterflies across disciplines is the wide interest in their complex and often conspicuous wing color patterns. Indeed, the basic color patterns for most butterfly wings are likely represented by the nymphalid groundplan^1^,^6^,^7^,^8^.

In contrast, biological studies that focus on lycaenid butterflies are scarce. Although lycaenid butterflies have wing color patterns as diverse as those of nymphalid butterflies and their small size makes them ideal study organisms, many lycaenid species are difficult to rear in a laboratory; these species may require the help of particular ant species to complete their life cycle or are not able to be maintained in small enclosures due to their strong flying ability. However, other lycaenid species do not require the assistance of ant species and have only a weak flying ability.

We have established a lycaenid model butterfly, the pale grass blue butterfly *Zizeeria maha*, that can be utilized for physiological, genetic, and environmental studies^9^. The natural history of this butterfly is well known^10^,^11^,^12^. It is multivoltine, completing one generation each month. Adults can be found from May to November in Honshu, including in the Tohoku and Kanto districts. Importantly, this species is small enough that hundreds or thousands of individuals may be reared in a small laboratory, without the assistance of any ant species. This species has already been used to study range expansion, its associated phenotypic plasticity and evolution^13^,^14^,^15^ and the developmental effects of mutagenesis^16^.

Following the massive environmental pollution caused by the release of radionuclides from the collapsed Fukushima Dai-ichi Nuclear Power Plant, the pale grass blue butterfly was used to monitor and examine the biological impacts of this pollution^17^,^18^,^19^,^20^,^21^,^22^. Forewing size, a proxy for body size^23^, of this butterfly was utilized as an indicator of the effects of environmental stressors. Adult butterflies collected from Fukushima City, Motomiya City, and Koriyama City (all within the Fukushima Prefecture) in the spring of 2011 had smaller forewing sizes than those collected from the northern and southern localities at the same time^17^. It has been demonstrated that small forewing size can be induced by external exposure to a cesium radiation source or internal exposure by ingesting contaminated foods^17^,^18^. Therefore, it is likely that the small forewing size detected only in Fukushima Prefecture in the spring of 2011 was caused by radioactive pollution^17^,^18^. However, to establish the pale grass blue butterfly as a standard environmental indicator species in Japan, it is necessary to study the natural forewing size distributions of this butterfly both spatially and temporally.

It has been known that animals, including arthropods, in cold environments (typically at high latitudes) have larger body sizes than those in warm environments. This observation is known as Bergmann’s rule^24^. Similarly, arthropod body size generally follows a temperature-size rule, which states that the body size is larger at low temperatures than at high temperatures^25^,^26^,^27^. Therefore, Bergmann’s rule and the temperature-size rule are often assumed to coexist or even to be synonymous. However, the generality of these rules are questioned by some authors because, at least in some arthropods and amphibians, the reverse of Bergmann’s rule is observed^23^,^28^,^29^. Furthermore, some mechanistic explanations of the temperature-size rule have been questioned^30^,^31^. To be sure, there are many laboratory rearing experiments that support the temperature-size rule, and its adaptive significance has been supported both experimentally and theoretically^32^,^33^. However, field observations of these rules that consider species-specific natural histories are still fragmentary. Two questions that remain unanswered are 1) does the forewing size of the pale grass blue butterfly vary with latitude and temperature, and 2) if so, how? These basic questions can be answered by field surveys and laboratory experiments (but not by theoretical calculations and other similar predictions). A better understanding of these questions would establish the pale grass blue butterfly as an important environmental indicator in Japan and provide a key to interpret the previous field data from the Fukushima nuclear accident.

To answer the questions, we first examined geographical variation in body (i.e., forewing) size of this species by comparing populations among prefectures in Japan. Butterflies were collected from several prefectures that covered the distribution of a subspecies, *Z. maha argia* (Ménétriès, 1857)^11^,^12^, from the northernmost prefecture (Aomori) to the southernmost prefecture (Kagoshima) (Supplementary Fig. 1; Supplementary Tables 1 and 2). In this way, the status of the Fukushima population was clarified in our study. Furthermore, we monitored seasonal changes in forewing size at a single locality over one year. We were also able to reproduce this field size variation at a single locality in the laboratory. These independent field and laboratory studies help to construct a coherent spatiotemporal picture of the body size of this butterfly in Japan. To our surprise, this species did not follow Bergmann’s rule. Instead, it followed the converse Bergmann’s rule but adhered to the temperature-size rule. Together, the present study not only provides valuable information about the body size variation of this species in Japan but also represents an important case study that can be used to simultaneously understand both geographical and temporal body size variation in a variety of species.

## Results

### Forewing size in the fall of 2012

**Figure 1:**
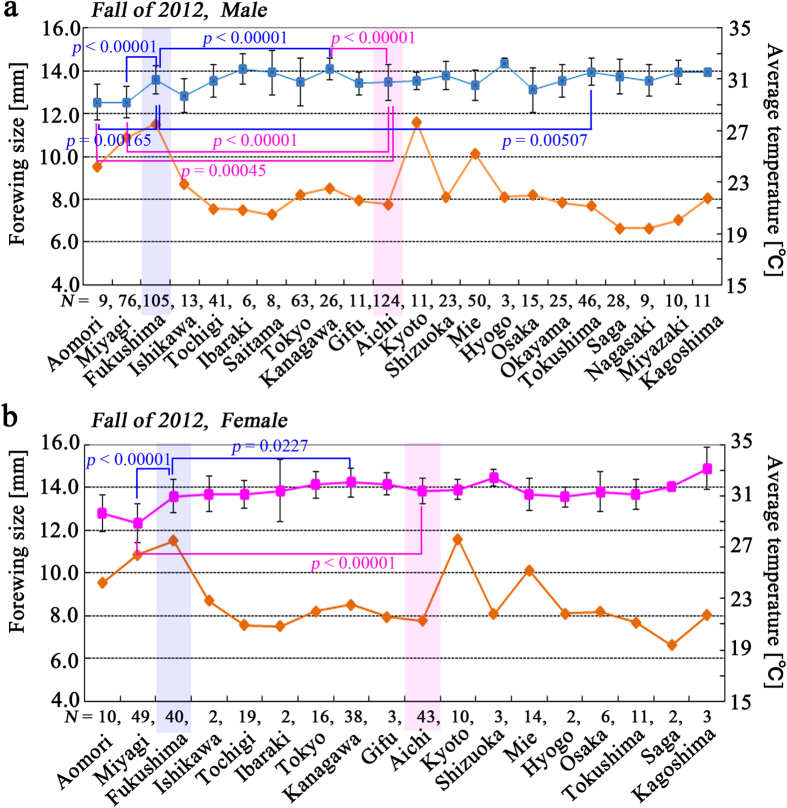
Forewing size distribution in the summer of 2012. Prefectures are aligned from northern (left) to southern (right) along the horizontal axis. Forewing sizes are shown in blue or pink square symbols, and average temperatures are shown in orange diamond symbols. Data points and error bars indicate mean ± SD. Prefectural populations from Fukushima and Aichi are shaded in blue and pink, respectively. Any prefecture that significantly differed (*p* < 0.05) from the Fukushima or Aichi populations is indicated with its *p*-value. Temperature data of Aomori, Miyagi, Fukushima, and Kyoto were deviated from the rest because the butterfly samples from these localities were collected in September. The samples from other localities were collected in October. (**a**) Males. (**b**) Females.

We first examined forewing size of males collected in the fall of 2012 from various prefectures (Fig. 1a). Multiple pairwise comparisons (i.e., Holm-corrected *t*-tests with pooled SD, performed here and in subsequent analyses) revealed that the Aomori population was significantly different from all other prefectural populations except the Miyagi, Ishikawa, and Osaka populations. Similarly, the Miyagi population was significantly different from all other populations except the Aomori, Ishikawa, and Osaka populations. Males in the Aomori and Miyagi populations had relatively small forewing sizes. Prefectural populations with relatively small forewing size may also include the Ishikawa population (which was significantly different from the Kanagawa, Shizuoka, and Tokushima populations) and the Osaka population (significantly different from the Kanagawa, and Tokushima populations).

The Kanagawa population was significantly different from 9 prefectural populations. Males from the Kanagawa population were larger than those in the other populations. The Hyogo, Ibaraki, Saitama, Kagoshima and Miyazaki populations, which were all significantly different from the Aomori and Miyagi populations, were also relatively large. Interestingly, the Aichi population was significantly larger than the Aomori and Miyagi populations but significantly smaller than the Kanagawa population (Fig. 1a). Similarly, the Fukushima population was larger than the Aomori and Miyagi populations but smaller than the Kanagawa and Tokushima populations (Fig. 1a). Other pairs of prefecture populations were mostly non-significant.

In females (Fig. 1b), the Miyagi population was significantly different from 12 of 17 populations, excluding 5 populations (the Aomori, Hyogo, Ibaraki, Ishikawa, and Saga populations) due to small sample sizes. Females from the Miyagi population had the smallest forewing size, and females from the Kanagawa population had the largest forewing sizes (significantly different from the Aomori, Miyagi, and Fukushima populations). The Aichi population was significantly different from the Miyagi population but not from the Kanagawa population (Fig. 1b). Again, the Fukushima population was significantly different from the Miyagi and Kanagawa populations (Fig. 1b), such that the Fukushima population was larger than the Miyagi population and smaller than the Kanagawa population. No other pairs of populations exhibited significant differences.

Although a size gradient was not discernable in this analysis, the fact that the two northernmost populations, the Aomori and Miyagi populations, were significantly smaller suggests that there is a size cline along a latitude or temperature gradient. Furthermore, the status of the Fukushima population and, to a lesser extent, the Aichi population in our analysis indicated that these populations may contain butterflies with the medial or representative size in Japan.

### Forewing size in the spring of 2013

We next examined the samples collected in the spring of 2013. In males, multiple pairwise comparisons revealed that the Miyagi population significantly differed from 5 other populations (the Aichi, Gifu, Kagoshima, Miyazaki, and Shizuoka populations) (Supplementary Fig. 2a). The Miyagi population was thus relatively small. The Wakayama population was relatively large but not statistically significant. In females, no significant difference in size was detected by ANOVA (*df* = 12, *F* = 1.8, *p* = 0.083) or multiple pairwise comparisons (*p *> 0.05 in all pairs) (Supplementary Fig. 2b).

### Latitudinal and temperature correlations in the fall of 2012

**Figure 2:**
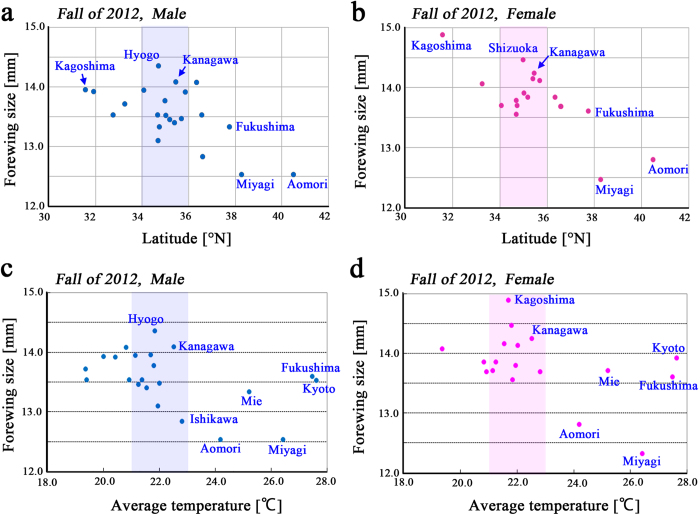
Scatter plot of the forewing sizes of butterflies collected in the fall of 2012. Shaded areas indicate high size variability within the latitudes between 34°N and 36°N and temperatures between 21 °C and 23 °C. (**a**) Scatter plot of male forewing size versus latitude. (**b**) Scatter plot of female forewing size versus latitude. (**c**) Scatter plot of male forewing size versus temperature. (**d**) Scatter plot of female forewing size versus temperature.

To test if there is a forewing size cline in relation to latitude, we made scatter plots and obtained Spearman’s and Kendall’s correlation coefficients for rank-transformed data and their associated *p*-values. In males, forewing size exhibited a weak inverse relationship to latitude (Spearman’s correlation coefficient *ρ* = −0.4795, *p* = 0.0280; Kendall’s correlation coefficient *τ* = –0.3625, *p* = 0.0182) (Fig. 2a). In females, forewing size exhibited a similar inverse relationship to latitude (*ρ* = –0.5075, *p* = 0.0367; *τ* = –0.3961, *p* = 0.0217) (Fig. 2b). In both sexes, the latitudinal band from 34°N to 36°N contained populations with various forewing sizes, which likely contributed to the small coefficients. Nonetheless, these results were largely in accordance with the converse Bergmann’s rule.

We examined if there was a forewing size cline related to temperature. Males exhibited small coefficients and high *p*-values (*ρ* = −0.4404, *p* = 0.0436; *τ* = −0.2876, *p* = 0.0610) (Fig. 2c). Females also exhibited small coefficients and high *p*-values (*ρ* = −0.3401, *p* = 0.1609; *τ* = −0.2244, *p* = 0.1934) (Fig. 2d). It is notable that forewing size varied greatly at temperatures between 21 °C and 23 °C in both sexes, suggesting that genetic differences among local populations arose in response to temperature or other environmental factors. The position of the Miyagi population was notable, as the sizes of both sexes were very different from the Fukushima population despite their similar temperatures and adjacent geographical locations.

### Latitudinal and temperature correlations in the spring of 2013

**Figure 3:**
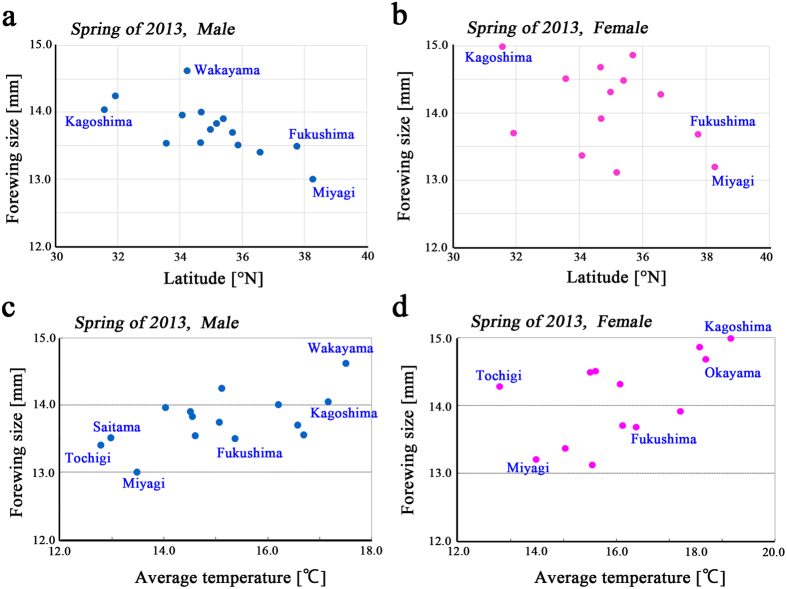
Scatter plot of the forewing sizes of butterflies collected in the spring of 2013. (**a**) Scatter plot of male forewing size versus latitude. (**b)** Scatter plot of female forewing size versus latitude. (**c**) Scatter plot of male forewing size versus temperature. (**d**) Scatter plot of female forewing size versus temperature.

We examined scatter plots of the relationship of latitude and forewing size for the samples collected in the spring of 2013. In males, a relatively large negative correlation coefficient was obtained (*ρ* = −0.7571, *p* = 0.0046; *τ* = −0.5810, *p* = 0.0025) (Fig. 3a), which is consistent with the converse Bergmann’s rule. In contrast, there was no clear correlation of these variables for females (*ρ* = −0.3571, *p* = 0.2160; *τ* = −0.256, *p* = 0.2224) (Fig. 3b).

Similarly, a positive correlation was obtained between temperature and forewing size in males (*ρ* = 0.5607, *p* = 0.0359; *τ* = 0.3714, *p* = 0.0563) (Fig. 3c) and females (*ρ* = 0.5989, *p* = 0.0380; *τ* = 0.4359, *p* = 0.0381) (Fig. 3d). It is to be noted that the temperature range here (12 °C to 18 °C or 20 °C) is different from that in the fall of 2012 (18 °C to 28 °C).

### Forewing size in Miyagi and Fukushima Prefectures

**Figure 4:**
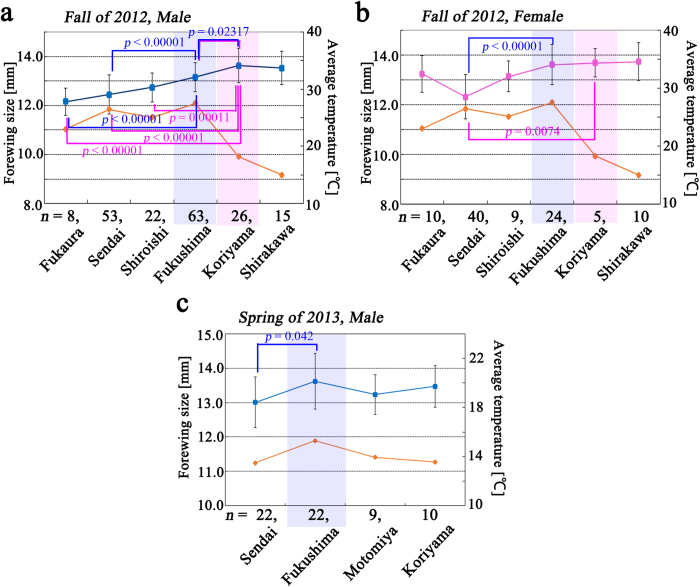
Forewing size distribution in Miyagi and Fukushima Prefectures. Local collection cities are aligned from northern (left) to southern (right). Fukaura City is in Aomori Prefecture, Sendai and Shiroishi Cities are in Miyagi Prefecture, and Fukushima, Koriyama, and Shirakawa Cities are in Fukushima Prefecture. Forewing sizes are shown in blue or pink square symbols, and average temperatures are shown in orange diamond symbols. Data points and error bars indicate mean ± SD. The populations found in Fukushima and Koriyama City are shaded in blue and pink, respectively. Any prefecture that significantly differed (*p* < 0.05) from the Fukushima or Koriyama populations is indicated with its *p*-value. (**a**) Males in the fall of 2012. (**b**) Females in the fall of 2012. (**c**) Males in the fall of 2013.

We examined forewing size differences at higher spatial resolution within Miyagi and Fukushima Prefectures. Instead of the prefecture-based comparisons above, local populations were independently treated based on sampling cities. We focused on data from Fukushima City and Koriyama City, in which small forewing sizes were recorded in 2011^17^. Multiple comparisons revealed that males from Fukushima City (Fukushima Prefecture) were highly significantly different from those of Sendai City (Miyagi Prefecture) (*p* < 0.00001) and Fukaura City (Aomori Prefecture) (*p* < 0.00001). We could not detect a significant difference between the populations of Fukushima City (Fukushima Prefecture) and Shiroishi City (Miyagi Prefecture) (Fig. 4a). However, the population of Koriyama City (Fukushima Prefecture) was highly significantly different from those of Shiroishi City, Sendai City, and Fukaura City. Females from both Fukushima City and Koriyama City were highly significantly different from those of Sendai City (Fig. 4b). Indeed, females from Sendai City were significantly different from those of all the other populations examined, except those of the Shiroishi City population.

In the spring of 2013, we again detected a significant difference between the male populations of Fukushima City and Sendai City (*p* = 0.042) (Fig. 4c). It is notable that the temperature in Fukushima Prefecture was high relative to the 4 cities examined in the spring of 2013, and forewing size was also relatively large. We could not examine females in the spring of 2013 due to small sample size.

### Average seasonal difference (from fall to spring) in forewing size in Japan

**Figure 5:**
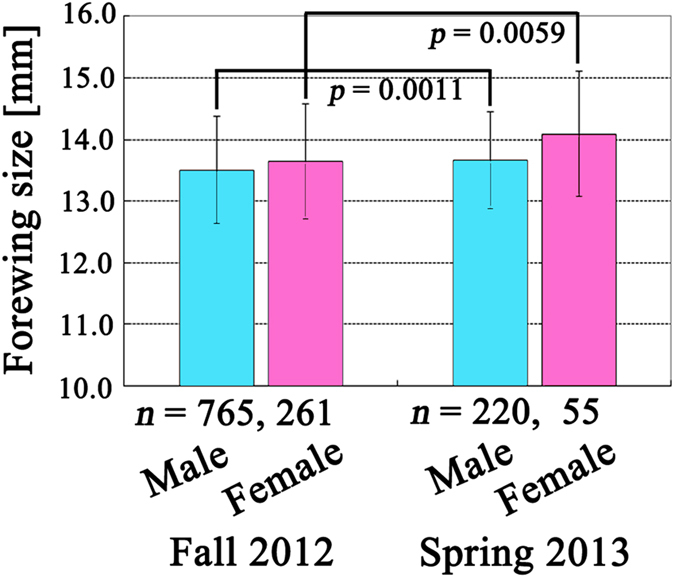
Forewing sizes in Japan in the fall of 2012 and the spring of 2013. Data points and error bars indicate mean ± SD.

We compared the forewing sizes of all samples (regardless of collection site or prefecture) collected in the fall of 2012 with those collected in the spring of 2013. In this way, we obtained *p* = 0.0011 for males and *p* = 0.0059 for females using unpaired two-sided *t*-tests (Fig. 5). Although there was considerable overlap in the standard deviations between the fall and spring, samples from the spring were significantly larger than those from the fall. This seasonal difference in forewing size has already been qualitatively documented for this species^10^−^12^. It is likely that forewing size negatively responds to temperature, which is in accordance with the temperature-size rule, although it is important to note that this result was obtained when ignoring geographical (latitudinal) information.

### Seasonal changes in forewing size over one year at a single locality

**Figure 6:**
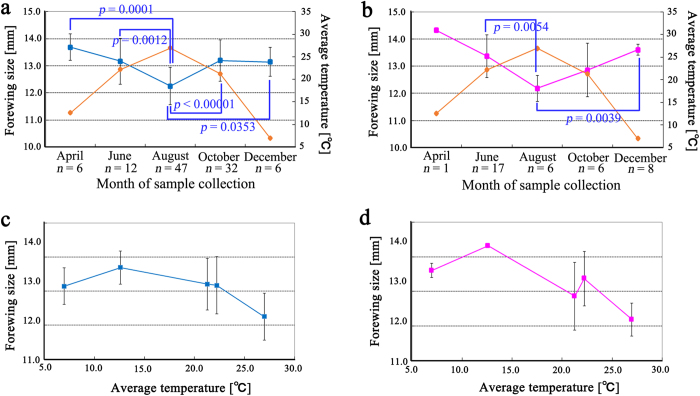
Seasonal forewing size changes at a single collection locality. Forewing sizes are shown in blue or pink square symbols, and average temperatures are shown in orange diamond symbols. Data points and error bars indicate mean ± SD. (**a**,**b**) Temporal changes in males and females. Month of collection is shown on the horizontal axis. Any month that significantly differed (*p* < 0.05) from August is indicated with its *p*-value. (**c**,**d**) Temperature dependence in males and females. Data are identical to those shown in (**a**,**b**).

To examine seasonal forewing size changes at a single locality, which may indicate the temperature-dependence of body size, we sampled the pale grass blue butterfly from Kirishima City, Kagoshima Prefecture, every other month from April to December 2013. In males, statistically significant differences were detected between April and August (*p* = 0.0001), between June and August (*p* = 0.0012), between August and all other time points (*p* < 0.05) (Fig. 6a). August samples were smaller than all other samples, and no other pairs of months exhibited significant differences. Likewise, in females, significant differences were found between August and June (*p* = 0.0054) and between August and December (*p* = 0.0039) (Fig. 6b). April samples were excluded due to a small sample size (*n* = 1). No other pairs of months exhibited significant differences.

In summary, forewing size was smallest in both sexes in August and largest in April and December. This pattern opposes that of temperature, which is highest in August and lowest in December and April (Fig. 6a,b). When the forewing size was plotted against temperature, an increasing size cline from 7 °C to 12.6 °C and a decreasing size cline from 12.6 °C to 27.0 °C were observed (Fig. 6c,d). It is likely that at least the Kirishima population of this species followed the temperature-size rule.

### Forewing size changes at different temperatures in the laboratory

**Figure 7:**
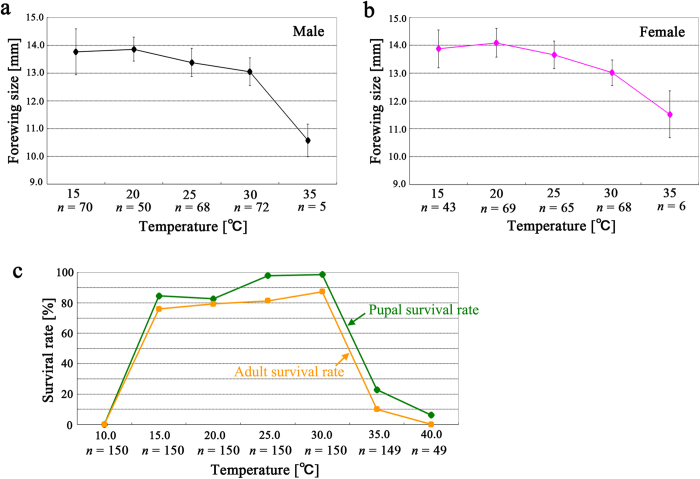
Temperature dependence of forewing size in a laboratory experiment. Data points and error bars indicate mean ± SD. (**a**) Males. (**b**) Females. (**c**) Surviv**a**l rate in response to temperature.

To examine how temperature affects forewing size, we reared larvae under different temperature conditions (from 10 °C to 40 °C, at intervals of 5 °C) in the laboratory. For this experiment, we utilized larvae that were obtained from females collected from Kirishima City, Kagoshima Prefecture. No individuals of both sexes were able to survive to the adult stage at 10 °C and 40 °C. Our results followed the temperature-size rule from 15 °C to 35 °C: at lower temperatures, forewing sizes were larger (Fig. 7a,b). At 15 °C, the forewing size was smaller than at 20 °C, but this was not statistically significant (*p* = 0.36 for males; *p* = 0.073 for females).

Individuals of both sexes reared at 20 °C and those at 15 °C were both significantly larger than those reared at any other temperature (*p* < 0.001). Indeed, size differences between all pairs except between 20 °C and 15 °C were highly significant in males (*p* = 0.002 between 25 °C and 30 °C, and *p* < 0.001 in all other pairs). For females, size differences between 15 °C and 20 °C and between 15 °C and 25 °C were not significant (both *p* = 0.073), but all other pairs were highly significant (*p* < 0.0001).

Survival rates based on eclosion (calculated as the number of individuals normally eclosed divided by the number of larvae reared) were all greater than 70% from 15 °C to 30 °C (Fig. 7c). Similarly, survival rates based on pupation (calculated as the number of individuals normally pupated divided by the number of larvae reared) were all greater than 80% from 15 °C to 30 °C (Fig. 7c). These results demonstrated that physiologically adequate temperatures for this butterfly are between 15 °C and 30 °C. Survival rates at 35 °C were relatively low (Fig. 7c), and small forewing size at this temperature may be considered pathological.

## Discussion

In this paper, we report geographical (i.e., latitudinal or spatial) and seasonal (i.e., temporal) size distributions of the pale grass blue butterfly *Z. maha argia* in Japan. We obtained 1,026 adults (765 males and 261 females) from 22 prefectures in the summer of 2012, and 275 adults (220 males and 55 females) from 15 prefectures in the spring of 2013 from Japan’s mainland. Our sampling covered almost the entire distribution of this species.

We detected statistically significant forewing size differences among prefectural populations. Notably, the populations in the northernmost prefectures, Aomori and Miyagi, exhibited smaller forewing sizes. This small size in Aomori and Miyagi Prefectures may be explained by their proximity to the northern range margin of this species, where a compromise of physiological function may be imposed. In addition to the Aomori and Miyagi populations, we found that some populations had statistically larger or smaller forewings. Notably, the Kanagawa population had larger forewings than many other populations, though the cause of this difference is unclear. These unexplained differences might be caused by geographical isolation or by a founder effect, as *Z. maha* populations could be easily isolated as a result of the species’ relatively poor flying ability. It is important to note that some prefectural populations, such as the Fukushima and Aichi populations, had larger forewings than the Aomori and Miyagi populations and were smaller than the Kanagawa population. The Fukushima and Aichi populations may be considered to have the medial or representative forewing size of all populations within Japan.

Importantly, we obtained a reasonable correlation between latitude and forewing size, especially for females in the fall of 2012 and males in the spring of 2013. The causes of these sexual and seasonal differences are unclear, but they might be the result of the natural history of this butterfly. We conclude that there is a latitudinal inverse cline of forewing size throughout Japan’s mainland. We confirmed that local temperature was also correlated with forewing size in a manner similar to the latitudinal trends in the spring of 2013. The temperature cline was largely non-significant in the fall of 2012. Between 34°N and 36°N and between 21 °C to 23 °C, many localities are located in these ranges, and forewing size varied considerably. This result could mean that *Z. maha* is adapted to latitude (which incorporates adaptation to temperature and other environmental factors) over many years, but not to temperatures within a single year. Moreover, it is important to note that individuals collected in the fall of 2012 (i.e., September and October 2012) had no prior experience with low temperatures, as they were produced approximately one month before they were caught. Therefore, it is reasonable to conclude that the local forewing size is primarily a genetically fixed trait for this butterfly species.

When examining the subpopulations of Miyagi and Fukushima Prefectures, it is evident that the populations of Fukushima City and Koriyama City (the Fukushima Prefecture) have larger forewings than those of the higher-latitude Sendai City and Shiroishi City (the Miyagi Prefecture). This prefectural boundary nearly follows the geographical features of this region, corresponding to a relatively narrow plain between the northern end of the Abukuma Mountains at the east coast and the Ohu Mountains on the west side.

Previously, we found small male forewing sizes in the spring of 2011 in Fukushima City, Motomiya City, and Koriyama City but not in a northern locality (Shiroishi City) or southern localities (Ibaraki and other prefectures). We have performed field surveys twice each year since 2011, but we did not observe this phenomenon after the spring of 2011^17^,^19^,^22^. In light of the results of this study, we conclude that the small forewing size detected in the spring 2011 in Fukushima Prefecture was caused by environmental stress that was imposed by an unusual event (likely the Fukushima nuclear accident) covering a relatively large geographical range of Fukushima Prefecture.

Despite the latitudinal forewing size cline discussed above, we demonstrated a seasonal forewing size change between the fall of 2012 and the spring of 2013 across all populations in Japan, broadly illustrating that forewing size in the fall population is smaller than that of the spring population. This observation has been qualitatively reported by Japanese lepidopterists but initially seems contradictory with the latitudinal cline (or inverse temperature cline) identified in this study. Bimonthly surveys of the Kirishima population over one year at a single locality revealed that forewing size indeed changes over a year and depended on the month of collection (and, thus, temperature). The higher the temperature, the smaller the forewing size. This pattern demonstrated that *Z. maha argia* temporally follows the temperature-size rule at a single locality. Interestingly, samples from December (i.e., the month with the lowest temperature) did not show the largest forewings; this result may indicate that low temperatures in December may work as an extreme stressor that is beyond physiological adaptation. Including this aspect, we largely reproduced the results of the field size changes in the rearing experiment in the laboratory using the same Kirishima population. We can thus conclude that the size changes over time that were observed in the field-caught samples were largely attributable to temperature changes. Therefore, this size variation in response to temperature is a physiologically plastic, not genetically fixed, trait.

**Figure 8:**
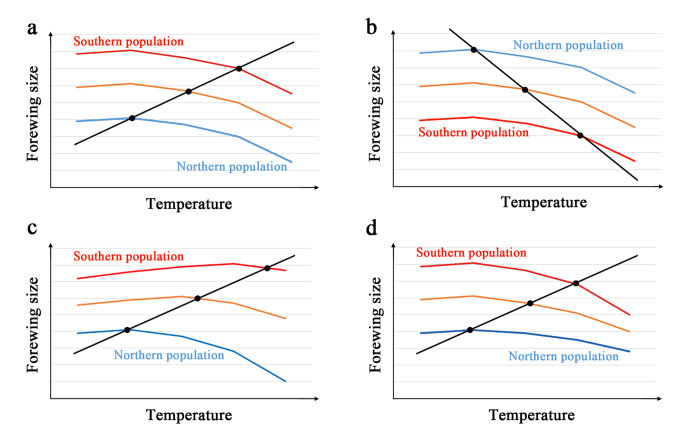
Models for possible relationships between the temperature-size rule and the converse Bergmann’s rule. (**a**) Coexistence of the temperature-size rule and the converse Bergmann’s rule. This model is in accordance with the results of this study. Southern populations are genetically larger than northern populations, although they similarly physiologically respond to temperature. (**b**) Coexistence of the temperature-size rule and Bergmann’s rule. This model is not in accordance with the results of this study. (**c**) A model modified from (**a**) in which peaks are located at different temperatures in different populations. (**d**) Another model modified from (**a**) in which the degree of physiological size changes differs among populations.

How can these latitudinal (genetic) and temporal (physiological) size changes be explained? Both types of size changes are correlated with temperature, but the former is a consequence of temperature responses over many years. The low temperature in winter season may be responsible, as it works as a selection pressure each winter. The latter is a consequence of temperature responses at a given larval period. Possible differences between these types of size changes are summarized in Supplementary Table 3. Both the converse Bergmann’s rule and the temperature-size rule can be understood without contradiction, as shown graphically in Fig. 8, where body size responses to temperature are population-dependent. Southern populations are overall larger than the northern populations, but because their temperature-sensitive size spectra closely resemble in this model, the reverse of Bergmann’s cline emerges (Fig. 8a). A model with a Bergmann’s cline is also possible (Fig. 8b); although this pattern is often assumed in size biology, such evidence was not obtained in the present study. A population-dependent temperature response has been shown in the cabbage white butterfly, *Pieris rapae*^34^. A balance between genetic and physiological responses may determine the final body size of a given individual.

In this discussion, we have implicitly assumed that the range of size plasticity (i.e., the reaction norm) between the largest and smallest individuals at a given locality does not differ in any local populations in Japan. However, the plasticity range may vary among local populations, as there are considerable temperature differences between summer and winter and between day and night in the northernmost regions but not in the southernmost regions. Local populations may have genetically adapted to environmental temperatures in terms of their physiological functions. In this sense, comparisons between the northernmost and southernmost populations may be informative in the future. Different populations may exhibit peak sizes at different temperatures (Fig. 8c) or may show different response profiles to temperature changes (Fig. 8d). Furthermore, the degree of size change appeared to be smaller in females than in males. This sexual difference may also vary among local populations, although its significance is not clear.

The reason why genetic and physiological responses are opposite in *Z. maha argia*, and likely also in other ectotherms, is somewhat enigmatic. We assert that life history is not different throughout Japan. However, coping with a relatively long winter season and short summer season at high latitudes may shape a geographical cline. It is known that body size responses to temperature in Orthoptera are likely dependent on voltinism and generation time^26^,^28^,^31^,^35^,^36^. We previously speculated that multivoltine insects with a short generation time, such as the pale grass blue butterfly, should have body sizes that are not likely to be affected by the timing of the winter season^18^. However, we now believe that rapid development to an adult form is important for northern populations of the pale grass blue butterfly, because of the relatively quick transition from summer to winter that may lead to genetic assimilation of the smaller size trait, resulting in the converse Bergmann’s rule. More frequent field sampling attempts may be necessary to solve this issue. A possible contribution of the host plant’s variation and natural history (in response to temperature and latitude) to the body size of the butterfly, if any, may be clarified in the future. It is interesting to investigate whether the case of *Z. maha* can be explained by latitudinal compensation and, if so, which model of gradient variation (i.e., countergradient or cogradient variation) is more appropriate for this species^37^,^38^.

In the tobacco hornworm, *Manduca sexta*^39^,^40^, and in *Drosophila*^41^, developmental time is affected by temperatures via hormonal changes. Similar hormonal factors may be responsible for forewing size in the pale grass blue butterfly; these factors will be examined in the future to support our interpretations, discussed above. Furthermore, it has been proposed that temperature-related hormones, such as ecdysteroids and cold-shock hormones (CSHs), function in pupae of nymphalid butterflies to determine wing color patterns^15^,^42^,^43^. Their potential functions in body size determination await further study. Furthermore, it has been proposed that butterfly color pattern evolution is at least partially driven by phenotypic plasticity in response to environmental changes^13^,^14^,^15^,^42^,^44^. Plastic expression of color patterns and wing size may be coordinated developmentally by cell proliferation to adjust the number of cells and by cell growth to adjust cell size^45^.

Finally, it is to be noted that the body size of the populations in the Ryukyu Archipelago, designated as *Z. maha okinawana* (Matsumura, 1929), is smaller than that on Japan’s mainland, *Z. maha argia* (Ménétriès, 1857)^11^,^12^. In this sense, *Z. maha* may geographically follow Bergmann’s rule as a species. We believe that body size rules and their associated discussion in biology are often oversimplified based on fragmentary results. At least for *Z. maha*, Bergmann’s rule, the converse Bergmann’s rule, and the temperature-size rule are all correct, depending on the system of interest. Field collection of *Z. maha* from the entire global distribution is required to accurately understand the whole picture of the body size distribution in this species. Whether *Z. maha okinawana* itself follows Bergmann’s rule or the converse rule within its distribution is an interesting question to be addressed in the future.

In conclusion, there was a clear geographical forewing size cline of *Z. maha argia* on Japan’s mainland. However, smaller forewing size was detected in the northernmost prefectures, Miyagi and Aomori. In contrast, there were marked seasonal forewing size changes that followed the temperature-size rule in this species at a single local population. Moreover, forewing size was shown to be dependent on rearing temperatures, as predicted by the temperature-size rule. These results provide basic geographical and physiological data on forewing size of the pale grass blue butterfly in Japan that can facilitate the use of this butterfly as an environmental indicator. Furthermore, this study confirmed our previous conclusion, that is, that the small forewing size of the pale grass blue butterfly in Fukushima Prefecture in the spring of 2011 was likely due to the Fukushima nuclear accident. Biological impacts of global warming may also be assessed by the study of the pale grass blue butterfly in the future.

## Methods

### Ethics statement

The pale grass blue butterfly, *Z. maha*, can be studied without any specific permission in Japan. This species is the most common and abundant butterfly in Japan (except in Hokkaido) and is distributed in both rural and urban regions^10^,^11^,^12^,^18^,^20^,^46^, making this species especially suitable for environmental studies^17^,^18^,^19^,^20^,^21^,^22^. It is not an endangered or protected species.

### Butterflies

The pale grass blue butterfly, *Z. maha* (Kollar, 1844) (Lepidoptera, Lycaenidae), was used in this study. The population found on Japan’s mainland is the subspecies *Z. maha argia* (Ménétriès, 1857)^11^,^12^. This population is distributed in Honshu, Shikoku, and Kyushu, but not in Hokkaido, from Aomori Prefecture in the north (approximately 30°N) to Kagoshima Prefecture (approximately 40°N) in the south. This butterfly repeats life cycle approximately 7 times a year and overwinters as larvae, which probably start growing slowly at the beginning of April or at the end of March in the Tohoku and Kanto districts^10^,^11^,^12^. It is monophagous, and its host plant *Oxalis corniculata* is distributed all over Japan including Hokkaido.

### Sample collection

To examine the influence of geography on forewing size of populations from the three main islands of Japan, adult butterflies were sampled from 59 sites (22 prefectures) in the fall (September and October) of 2012 and 45 sites (15 prefectures) in the spring (April and May) of 2013 (Supplementary Fig. 1; Tables S1, S2). Two sampling periods were used because this species is known to exhibit seasonal polyphenism^10^,^11^,^12^. Sample collection was mostly conducted by volunteers from local insect clubs and others who appreciated the scientific and social importance of our research. In statistical analyses, butterfly samples were categorized by their prefecture of collection.

To examine how forewing size changes over one year, adult butterflies were sampled from Kokubu Undou Park and Noguchi Bridge over the Amori River, Kirishima City, Kagoshima Prefecture, in April, June, August, October, and December 2013. These sampling sites are separated by only a few kilometers, and they were thus considered to be a single collection locality. We obtained offspring from these Kirishima females, and the offspring were used in the temperature experiments.

### Forewing size measurements

Forewing size was measured in digital images using a SKM-S30A-PC digital microscope and its associated software, SK Measure (Saitou Kougaku, Yokohama, Japan). We measured the distance from the wing base to the marginal band at the end of the M_1_ vein in the left ventral forewing. Individuals with damaged wings were excluded from measurements. When only a single male or female was obtained from a given prefecture, the sample was excluded from analyses.

### Field temperature records and latitude

We obtained temperature records for the prefectures of the collection sites from the Japan Meteorological Agency (www.jma.go.jp). We used the temperature at the prefectural capital city as the temperature of the collection site. We also used the temperature records of Kagoshima Aviation Weather Station, Kirishima City, Kagoshima Prefecture (www.jma-net.go.jp/kagoshima-airport) as the reference temperature when assessing the bimonthly changes in forewing size. We first obtained daily temperature records (mean temperature) from the earliest to the latest dates for each prefectural population. Additionally, we obtained temperature data for the 30 days before the earliest collection date, as generation time is approximately 30 days for this species. We also assumed that the overwintering larvae start growing slowly at the beginning of April, one month before the emergence of adults. We then calculated the average values of these daily temperature records. The resultant values were used as the temperatures for collected samples. Thus, the entire developmental stages were covered for the summer collection, and for the spring collection, the stages of growing larvae and pupae were covered. In the fall collection, most samples were collected in October, but those of Aomori, Miyagi, Fukushima, and Kyoto were collected in September. Thus, temperature data on these localities were deviated from the rest.

We did not use day-degrees, which are calculated based on the assumption that a given species has a single fixed threshold for growth, because of the following reasons. First, we do not know an accurate threshold for the pale grass blue butterfly. Second, we have preliminary evidence that northern and southern populations of the pale grass blue butterfly have different sensitivity to cold and hot temperatures. That is, the threshold is not a fixed value, and it is also likely a product of adaptive evolution to local environment. We used the latitudes of a prefecture’s capital city as the prefectural latitude. Use of latitude is based on the idea that it may be a factor that integrates many primary environmental factors such as temperature and photoperiod.

### Temperature experiments

Egg collection and rearing procedures are described elsewhere^9^. We collected adult butterflies in April 2013 in the area surrounding Noguchi Bridge over the Amori River, Kirishima City, Kagoshima Prefecture. A total of 5 males and 6 females were confined in a single cage consisting of a cubic glass container (300 mm per side). The resulting eggs were randomly divided into 5 groups and were reared in incubators set at either 40 °C, 30 °C, 25 °C, 20 °C, or 15 °C under a long-day photoperiod (16L:8D). The eggs that were reared in incubators set at 35 °C and 10 °C were collected from 6 males and 10 females obtained in October 2013 at the same site and confined in the cage described above. Incubator temperature fluctuations were ±1 °C. We set two pots of flowers, dahlberg daisy (*Thymophylla tenuiloba*; preferred) or butter daisy (*Melampodium paludosum*), in each cage for nectar sucking. We also put two pots of the host plant, *Oxalis corniculata*, in a cage for ovipositioning. After a large number of eggs were deposited on the host plant’s leaves, the host plant was replaced. We put a plastic bag over the entire potted host plant with eggs until we could confirm that first-instar larvae had hatched by visually observing that leaves had been eaten. We then transferred larvae to a plastic container containing leaves. Larvae were reared at ambient temperature (approximately 27 °C) until they grew to the second or third instar, after which they were placed in an incubator at the temperature specific to their treatment. Light was set at 18L:6D. Larvae and pupae were kept in incubators until eclosion.

### Statistical analysis

To compare forewing size differences among the prefectural populations, we used R statistical software, version 3.0.2 (R Foundation for Statistical Computing, Vienna, Austria). We conducted ANOVAs, multiple pairwise comparisons (Holm-corrected *t*-test with pooled SD), and unpaired *t*-tests. Two-sided *p*-values were reported. Size differences were considered to be statistically significant when *p* > 0.05. Mean and standard deviation values were calculated using the AVERAGE and STDEV functions in Microsoft Excel, respectively. Scatter plots were made using Microsoft Excel. Spearman’s correlation coefficients (*ρ*), Kendall’s correlation coefficients (*τ*), and two-sided *p*-values were obtained using JSTAT 13.0 (Yokohama, Japan). Because body size (including the wing size) is known to differ between the sexes in this species, males and females were tested separately in all analyses.

## Additional Information

**How to cite this article**: Taira, W. *et al*. Body size distributions of the pale grass blue butterfly in Japan: Size rules and the status of the Fukushima population. *Sci. Rep*. **5**, 12351; doi: 10.1038/srep12351 (2015).

## Supplementary Material

Supplementary Information
