# Supplementary material for: Body size distributions of the pale grass blue butterfly in Japan: Size rules and the status of the Fukushima population
Source: Sci Rep. 2015 Jul 22;5:12351. doi: 10.1038/srep12351 (PMC4510528; doi:10.1038/srep12351)
Supplement: Supplementary Information [file srep12351-s1.pdf]

Scientific Reports

Supplementary Information

Body size distributions of the pale grass blue butterfly in Japan: size rules and the status of the Fukushima population

Wataru Taira*, Mayo Iwasaki*, and Joji M. Otaki*

The BCPH Unit of Molecular Physiology,
Department of Chemistry, Biology and Marine Science,
Faculty of Science,
University of the Ryukyus,
Nishihara, Okinawa 903-0213,
Japan

*These authors contributed equally.

Correspondence and requests for materials should be addressed to J.M.O. (otaki@sci.u-ryukyu.ac.jp)

Supplementary Figure 1
Supplementary Figure 2
Supplementary Table 1
Supplementary Table 2
Supplementary Table 3

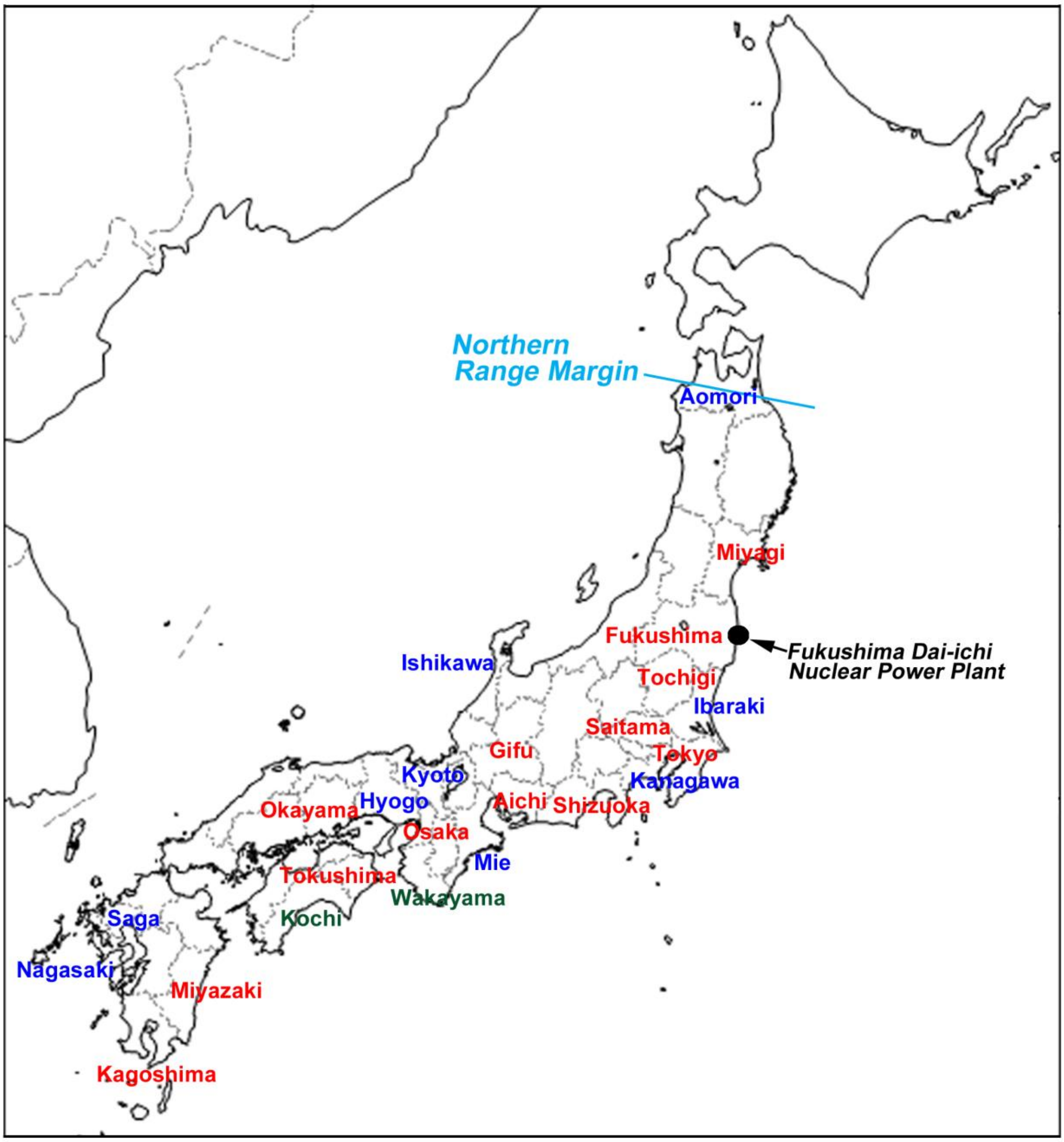

Supplementary Figure 1. Prefectures in Japan from which samples were collected. Prefectural boundaries are shown in broken lines. Prefectures from which samples were obtained both in the fall of 2012 and in the spring of 2013 are shown in red. Prefectures from which samples were obtained only in the fall of 2012 are shown in blue. Prefectures from which samples were obtained only in the spring 2013 are shown in green. Northern range margin crossing Aomori prefecture is indicated by a blue line. Location of the Fukushima Dai-ichi Nuclear Power Plant is indicated by a black dot. An original copyright-free blank map was obtained from “the blank map specialty shop” (www.freemap.jp). Prefecture names and other annotations were written by the authors.

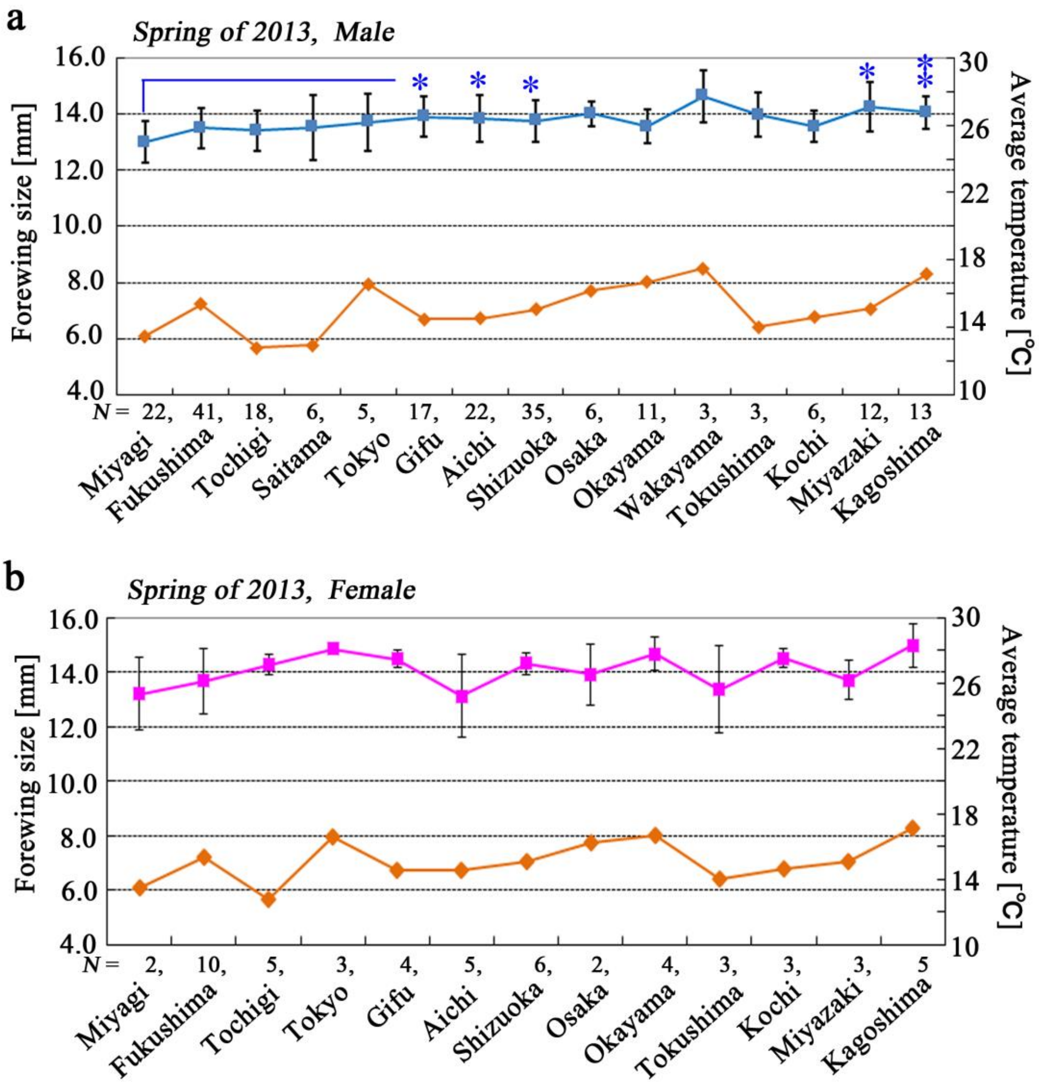

Supplementary Figure 2. Forewing size distribution in the spring of 2013. Prefectures are aligned from northern (left) to southern (right) ones in horizontal axis. Forewing sizes are shown in blue or pink square symbols, and average temperatures are shown in orange diamond symbols. Data points and error bars indicate mean ± SD. (**a**) Males. Asterisks indicate significant difference from the Miyagi population in multiple pairwise comparison (*, $p < 0.05$; **, $p < 0.01$). (**b**) Females. No significant differences were found in multiple pairwise comparisons.

Supplementary Table 1. Collection sites in the fall of 2012.

Prefecture	Collection sites
Aomori (*N* = 19)	[1] WeSPa Tsubakiyama, Nabeishi, Henashi, Fukaura-machi, Nishitsugaru-gun (*n* = 19)
Miyagi (*N* = 124)	[1] Sendai-Ohashi, Sendai-shi (*n* = 93) [2] Shiroishi Field & Track, Shiroishi-shi (*n* = 31)
Fukushima (*N* = 145)	[1] Aza-Hinata, Oniuta, Nishida-machi, Koriyama-shi (*n* = 15) [2] Aza-Kannozawa, Oniuta, Nishida-machi, Koriyama-shi (*n* = 16) [3] Nanko Park, Nishida-machi, Shirakawa-shi (*n* = 25) [4] Omori, Fukushima-shi (*n* = 36) [5] Hanamiyama, Fukushima-shi (*n* = 35) [6] Matsukawa PA (Parking Area), Hanamiyama, Fukushima-shi (*n* = 16) [7] Aza-Kashima, Oaza-Shobuya, Ono-machi, Tamura-gun (*n* = 2)
Ishikawa (*N* = 15)	[1] Rokugozaki, Noroshi-machi, Suzu-shi (*n* = 11) [2] Kodomari, Misaki-machi, Suzu-shi (*n* = 4)
Tochigi (*N* = 59)	[1] Yamato, Utsunomiya-shi (*n* = 5) [2] Kakashinosato, Nishiyamada, Ohira-machi, Tochigi-shi (*n* = 4) [3] Hanyuda, Mibu-machi (*n* = 5) [4] Mikamoyama Park, Sano-shi (*n* = 2) [5] Nakatomatsuri-cho, Utsunomiya-shi (*n* = 40) [6] Watarase Yusuichi, Tochigi-shi (*n* = 3)
Ibaraki (*N* = 8)	[1] Kamiizushima, Bando-shi (*n* = 4) [2] Ono, Takahagi-shi (*n* = 4)
Saitama (*N* = 9)	[1] Mizuno, Sayama-shi (*n* = 9)
Tokyo (*N* = 79)	[1] Chiyoda, Chiyoda-ku (*n* = 34) [2] Tachikawa Park, Shibasaki-cho, Tachikawa-shi (*n* = 2) [3] Tamagawa-Ryokuchi Sports Park, Nishirokugo, Ota-ku (*n* = 25) [4] Otsuka-Sekijou, Hachioji-shi (*n* = 14) [5] Inogashira Park, Gotenyama, Musashino-shi (*n* = 4)
Kanagawa (*N* = 64)	[1] Hiro-machi, Minami-Ashigara-shi (*n* = 2) [2] Numashiro, Odawara-shi (*n* = 9) [3] Haneo, Odawara-shi (*n* = 4) [4] Tsuchiya, Hiratsuka-shi (*n* = 11) [5] Ogikubo, Odawara-shi (*n* = 38)
Gifu (*N* = 14)	[1] Hime-cho, Tajimi-shi (*n* = 14)
Aichi (*N* = 166)	[1] Imaike, Chikusa-ku, Nagoya-shi (*n* = 7) [2] Takahata Park, Nakagawa-ku, Nagoya-shi (*n* = 10) [3] Arako Park, Nakagawa-ku, Nagoya-shi (*n* = 12) [4] Tsurumai Park, Showa-ku, Nagoya-shi (*n* = 20) [5] Kawana Park, Syowa-ku, Nagoya-shi (*n* = 22) [6] Tamano-cho, Kasugai-shi (*n* = 17) [7] Oike, Kasugai-shi (*n* = 10) [8] Chitaboen Nishibuchima, Daikoji, Chita-shi (*n* = 48) [9] Taiho, Atsuta-ku, Nagoya-shi (*) [10] Atsutanishi-machi, Atsuta-ku, Nagoya-shi (*) [11] Teramae, Shimokawaguchi-cho, Toyota-shi (*n* = 3) [12] Hatsutachi Dam, Ishidoyama, kameyama-machi, Tahara-shi (*n* = 3) *Note: [9] and [10] together have 14 individuals whose exact collection sites are unknown.
Kyoto (*N* = 21)	[1] Aseri, Kyotanba-machi, Hunai-gun (*n* = 21)
Shizuoka (*N* = 26)	[1] Hanagura, Fujieda-shi (*n* = 9) [2] Yainaba, Fujieda-shi (*n* = 4) [3] Rengeji Park, Nyakuoji, Fujieda-shi (*n* = 10) [4] Matsushima-cho, Minami-ku, Hamamatu-shi (*n* = 3)
Mie (*N* = 63)	[1] Ioki, Ureshino-Yaoroshi-cho, Matsuzaka-shi (*n* = 63)
Hyogo (*N* = 5)	[1] Mizuno-cho, Suma-ku, Kobe-shi (*n* = 5)
Osaka (*N* = 21)	[1] Kyuhoji, Yao-shi (*n* = 4) [2] Osaka Prefecture University, Naka-ku, Sakai-shi (*n* = 5) [3] Osaka Prefecture University, Toyonaka-shi (*n* = 12)
Okayama (*N* = 26)	[1] Ioka, Mimasaka-shi (*n* = 10) [2] Tsujita, Mabi-cho, Kurashiki-shi (*n* = 3) [3] Fukushima, Kurashiki-shi (*n* = 13)
Tokushima (*N* = 57)	[1] Kurosaki, Muya-cho, Naruto-shi (*n* = 57)
Saga (*N* = 30)	[1] Livestock Experimental Station, Miyano, Yamauchi-cho, Takeo-shi (*n* = 30)
Nagasaki (*N* = 10)	[1] Togitsu-machi, Nagasaki-shi (*n* = 10)
Miyazaki (*N* = 10)	[1] Kogawa-machi, Nobeoka-shi (*n* = 10)
Kagoshima (*N* = 13)	[1] Haruyama-cho, Kagoshima-shi (*n* = 3) [2] Mugio, Yakushima-machi (*n* = 10)

Collection sites within a prefecture are numbered just for convenience. The numbers of individuals listed in this table contain both sexes. Refer to Fig. 1 for the numbers of males and females.

Supplementary Table 2. Collection sites in the spring of 2013.

Prefecture	Collection sites
Miyagi (*N* = 35)	[1] Sendai-Ohashi, Sendai-shi (*n* = 35)
Fukushima (*N* = 66)	[1] Araike Park, Koriyama-shi (*n* = 1) [2] Furusato-no-Kawa Park, Koriyama-shi (*n* = 11) [3] LAWSON Arai Branch, Motomiya-shi (*n* = 2)** [4] Janohana-Yurakuen Motomiya-shi (*n* = 6)** [5] Omori, Fukushima-shi (*n* = 14)*** [6] Hanamiyama, Fukushima-shi (*n* = 15)***<br>**Note: [3] and [4] have additional 8 individuals whose exact collection sites are unknown.<br>***Note: [5] and [6] have additional 9 individuals whose exact collection sites are unknown.
Tochigi (*N* = 24)	[1] Nakatomatsuri-cho, Utsunomiya-shi (*n* = 24)
Saitama (*N* = 7)	[1] Mizuno, Sayama-shi (*n* = 7)
Tokyo (*N* = 8)	[1] Hyogosima Park, Tamakawa, Setagaya-ku (*n* = 8)
Gifu (*N* = 61)	[1] Oribeno-Oka Park, Seianji, Kujiri, Izumi-cho, Toki-shi (*n* = 7) [2] Kokeizan Eihoji, Kokeizan-cho, Tajimi-shi (*n* = 54)
Aichi (*N* = 28)	[1] Owaki-cho, Toyohashi-shi (*n* = 1) [2] Unoya-cho, Toyohashi-shi (*n* = 1) [3] Akabane-Bunka-no-Mori, Tahara-shi (*n* = 4) [4] Masaki/Asahi, Kagiya-machi, Tokai-shi (*n* = 5) [5] Jokoji, Jokoji-cho, Seto-shi (*n* = 11)[6] Kamiida-Minami-machi, Kita-ku, Nagoya-shi (*n* = 2) [7] Komaki East Interchange, Akechi-cho, Kasugai-shi (*n* = 1) [8] Idaka Ryokuchi Park, Idaka-machi, Meito-ku, Nagoya-shi (*n* = 3)
Shizuoka (*N* = 44)	[1] Boze, Kosai-shi (*n* = 1) [2] JR Misakubo Station, Jitokata, Misakubo-cho, Tenryu-ku, Hamamatsu-shi (*n* = 4) [3] Tokoji, Shimada-shi (*n* = 2) [4] Sengen-cho, Aoi-ku (*n* = 6) [5] Akazawa, Aoi-ku (*n* = 16) [6] Kabuta Bus Stop, Ohara, Aoi-ku (*n* = 15)
Osaka (*N* = 8)	[1] Minamino, Shijonawate-shi (*n* = 3) [2] Shijonawa-shi (*n* = 4) [3] Kawata, Higasiosaka-shi (*n* = 1)
Okayama (*N* = 15)	[1] Kuga, Mimasaka-shi (*n* = 1) [2] Mimasaka-shi (*n* = 10) [3] Kashira Island, Bizen-shi (*n* = 4)
Wakayama (*N* = 4)	[1] Arahune Coast, Kushimoto-machi (*n* = 3) [2] Kandorizaki, Taiji-machi (*n* = 1)
Tokushima (*N* = 6)	[1] Aza-Shimoban, Kitokitagawa, Naka-machi, Naka-gun (*n* = 6)
Kochi (*N* = 9)	[1] Nishino, Noichi-cho, Konan-shi (*n* = 8) [2] Fukabuchi, Noichi-cho, Konan-shi (*n* = 1)
Miyazaki (*N* = 16)	[1] Shiroyama, Nobeoka-shi (*n* = 1) [2] Omaru-ohashi, Takanabe-cho (*n* = 2) [3] Honkoji, Nobeoka-shi (*n* = 7) [4] Noda, Nobeoka-shi (*n* = 5) [5] Kawachimyo Nekodani, Kitagawa-machi, Nobeoka-shi (*n* = 1)
Kagoshima (*N* = 19)	[1] Haruyama-cho, Kagoshima-shi (*n* = 2) [2] Shitogo, Yakushima-machi (*n* = 1) [3] Kurio, Yakushima-machi (*n* = 16)

Collection sites within a prefecture are numbered just for convenience. The numbers of individuals listed in this table contain both sexes. Refer to Supplementary Fig. 2 for the numbers of males and females.

Supplementary Table 3. Two opposing factors that determine the forewing size of the pale grass blue butterfly in Japan.

	Forewing size determinant	
Factors examined / ***Features***	***Latitudinal (Geographical, Spatial)***	***Seasonal (Temporal)***
Rule<br>(relation to temperature)	Converse Bergmann's rule<br>(proportional to temperature)	Temperature-size rule<br>(inversely proportional to temperature)
Temperature distribution	Geographical	Local
Duration	Long term	Short term
Response (adaptation)	Genetic adaptation	Physiological adaptation
Selection pressure	High?	Low?
Possible mechanism	Cell number (proliferation)?	Cell size (growth)?
Advantage in cold environment	Small body size is fast to become adults and to reproduce.	Large body size has large offspring size.
Disadvantage in cold environment	Small body size has small offspring size.	Large body size is slow to become adults and to reproduce.
